# Evaluation of muscle biopsy in late-onset GSDII patients before and after enzyme replacement therapy (ERT)

**DOI:** 10.1186/1471-2474-14-S2-P13

**Published:** 2013-05-29

**Authors:** R Violano, M Ripolone, V Lucchini, L Villa, M Sciacco, G Comi, P Tonin, M Filosto, S Previtali, T Mongini, L Vercelli, E Vittonatto, A Toscano, O Musumeci, E Barca, C Angelini, S Ravaglia, C Lamperti, M Mora, L Morandi, M Moggio

**Affiliations:** 1 Neuromuscular Unit - Fondazione I.R.C.C.S. Ca’ Granda, Ospedale Maggiore Policlinico, Dino Ferrari Centre, University of Milan, Milan, Italy; on behalf of the Italian group of GSDII

## Introduction

Glycogen storage disease, glycogenosis type II (GSDII), or Pompe disease (OMIM 23230), is an autosomal recessive lysosomal storage disorder that results from a deficiency in the acid alpha glucosidase (*GAA*) enzyme. The disease is characterized by progressive accumulation of lysosomal glycogen in various tissues, primarily in cardiac and skeletal muscles. The histopathological hallmarks in the muscle are fiber vacuolization and autophagy. GSDII is clinically classified as a severe infantile, or an attenuated, later-onset form affecting children and adults. Recombinant human GAA (rhGAA) is the only approved enzyme replacement therapy (ERT) available for the treatment of Pompe disease, and it is effective in infantile patients, whereas in adults, improvements are more variable among different patients. Our project aims to assess the effects of ERT in 19 late-onset patients using both biochemical and morphological (histological, histochemical, and immunohistochemical) evaluations of skeletal muscle biopsies before and after 6 months to 1 year on ERT.

## Methods

Using baseline and post-ERT muscle biopsy tissue, we monitored autophagy by immunohistochemical analysis of the following proteins belonging to vesicular pathways: EEA1 early endosome, LC3 autophagosomal marker, and LAMP-2 lysosomal marker. Quantitative evaluations of fiber diameter, CSA, the number of vacuolated fibers, the degree of glycogen accumulation, and the percentage of vacuolization in type I and type II fibers were also performed.

## Results

All patients clinically improved after ERT. Pre-treatment muscle biopsies showed a histopathologically divergent spectrum, ranging from almost normal morphology with very few scattered vacuoles, to severe vacuolar myopathy. Post-treatment muscle biopsies morphologically improved in two patients, whereas no significant histopathological modifications were seen in all the other subjects. Immunohistochemical analysis of the autophafagic pathways was very complex and showed variable binding of the three antibodies in both the first and the second biopsies.

## Conclusion

This study highlights the effectiveness of ERT in patients with adult onset GSDII and helps shed light on the role of autophagy in the pathogenesis of the disease.

